# The effectiveness of different down-regulating protocols on in vitro fertilization-embryo transfer in endometriosis: a meta-analysis

**DOI:** 10.1186/s12958-020-00571-6

**Published:** 2020-02-29

**Authors:** Xue Cao, Hong-yang Chang, Jun-yan Xu, Yi Zheng, Yun-gai Xiang, Bing Xiao, Xu-jing Geng, Li-li Ni, Xi-ying Chu, Shi-bo Tao, Yan He, Gen-hong Mao

**Affiliations:** 1 Reproductive Medical Center, The Second Affiliated Hospital of Zhengzhou University, Zhengzhou, 450014 China; 2 Wuhan Institute of Dermatology and Venerology, Wuhan, China; 3 Teaching Office, The Second Affiliated Hospital of Zhengzhou University, Zhengzhou, 450014 China

**Keywords:** GnRH agonist, Endometriosis, In vitro fertilization, Pregnancy

## Abstract

**Background:**

To investigate the effectiveness of the GnRH-a ultra-long protocol, GnRH-a long protocol, and GnRH-a short protocol used in in vitro fertilization-embryo transfer (IVF-ET) in infertile women with endometriosis.

**Methods:**

We searched PubMed, Embase, Web of Science, Cochrane Library, Elsevier Science Direct, OA Library, Google Scholar, China National Knowledge Infrastructure (CNKI), Wanfang Data Knowledge Service Platform, China Science and Technology Journal database, and the China Biology Medicine disc for randomized controlled trials (RCTs) and observational studies (non-RCTs) to evaluate the efficacy of the GnRH-a ultra-long protocol, GnRH-a long protocol, and GnRH-a short protocol in IVF-ET in infertile patients with endometriosis.

**Results:**

A total of 21 studies in compliance with the standard literature were included, and RCT and non-RCT studies were analyzed separately. This meta-analysis showed that the GnRH-a ultra-long protocol could improve the clinical pregnancy rate of infertile patients in RCT studies, especially in patients with stages III–IV endometriosis (RR = 2.04, 95% CI: 1.37~3.04, *P* < 0.05). However, subgroup analysis found the different down-regulation protocols provided no significant difference in improving clinical outcomes in patients with endometriosis in the non-RCT studies.

**Conclusion:**

This study suggests that the GnRH-a ultra-long protocol can improve the clinical pregnancy rate of the patients with stages III–IV endometriosis in RCT studies. Although it is generally believed that the results of RCT are more reliable, the conclusions of the non-RCT studies cannot be easily neglect, which let us draw conclusions more cautious.

## Background

Endometriosis, a common clinical gynecological disease in women of childbearing age, refers to the presence of functional endometrial tissue (gland and stroma) in parts of the body outside the uterus. Although the disease has a benign manifestation in morphology, it has clinical behaviors characterized by similar malignant tumors, such as planting, invasion, distant metastasis, and recurrence. The main symptoms of endometriosis are lower abdominal pain and dysmenorrhea, sexual discomfort, and infertility. Studies have shown that about 30–50% of women with endometriosis have infertility, and about 20–50% of infertility patients have endometriosis [1]. Endometriosis with infertility is thought to be multifactorial and impairs fertility directly by destroying the normal anatomy of the fallopian tube and ovary or indirectly through inflammatory reaction and oxidative damage to degrade the quality of oocytes [2–5]. Of course, male factors, such as sperm quality, are also important factors affecting women’s fertility [6]. With the development of assisted reproductive technology, IVF-ET has gradually become an important treatment for patients with infertility from endometriosis. Pituitary down-regulation is a key link in the IVF-ET process. Gonadotropin-releasing hormone agonist (GnRH-a) can play a competitive role in the pituitary gland and block its release of GnRH, thereby inhibiting the secretion of related hormones in the ovary and achieving the effect of pituitary down-regulation. In addition, GnRH-a can effectively prevent premature luteinization of follicles and improve the synchronization of follicular growth and development [7]. Furthermore, it can reduce the degree of inflammatory reaction, improve the pelvic microenvironment, and obtain high-quality eggs and embryos [7, 8].

With the continuous exploration of the down-regulating protocol, research into adopting different down-regulation protocols to implement IVF-ET-assisted pregnancy in infertility patients with endometriosis and improve the success rate already exists at home and abroad, but the clinical outcomes are still controversial. One study showed that the 3–6 month GnRH-a therapy might increase their clinical pregnancy rate by four times [9], but another study found that ultra-long protocol and long protocol provide no significant difference in improving clinical outcomes in patients with endometriosis [10]. Therefore, this paper systematically evaluated, through meta-analysis, the clinical studies of the effects of GnRH-a ultra-long, long-term, and short-term protocols in the treatment of IVF-ET in infertile patients with endometriosis. The aim was to screen out the optimal down-regulation protocol for patients with infertility from endometriosis.

## Methods

### Search strategy

We performed a literature search in PubMed, Embase, Web of Science, Cochrane Library, Elsevier Science Direct, OA Library, Google Scholar, China National Knowledge Infrastructure (CNKI), Wanfang Data Knowledge Service Platform, the China Science and Technology Journal database, and the China Biology Medicine disc. The keywords included “endometriosis”, “IVF-ET/in vitro fertilization”, “ART”, “GnRH-a”, “ultra-long”, “prolonged”, “short”, “project”, and “protocol”. All titles and abstracts were read individually, and the literatures that clearly did not meet the inclusion criteria were screened out. Note-Express software (version 3.2, China) was then used to remove duplicates, and those that might meet the inclusion criteria were further screened by reading the full text. If the data was incomplete and the author could not be reached, the document was discarded. The review was registered in PROSPERO (CRD42019139831).

### Inclusion and exclusion criteria

To avoid selection bias, studies that met the following criteria were included in this meta-analysis: (1) clinical study of the efficacy of IVF/intracytoplasmic sperm injection (ICSI)-ET in the treatment of endometriosis infertility patients with GnRH-a ultra-long protocol, GnRH-a long protocol, and GnRH-a short protocol, including cohort studies and randomized controlled trials with no limit for language; (2) subjects of study were women diagnosed with endometriosis by laparoscopy, laparotomy, or transvaginal aspiration of the ovarian endometrial cyst combined with pathology and those who received IVF/ICSI-ET for infertility after surgery; diagnostic criteria according to the American Society for Reproductive Medicine (ASRM) classification; (3) studies that adopted the following controlled ovarian stimulation (COS) protocol: GnRH-a ultra-long protocol, GnRH-a long protocol (long-term or short-acting), and GnRH-a short protocol.

Exclusion criteria were as follows: (1) Comparison of the aforementioned three protocols with other COS protocols (such as modified GnRH-a ultra-long protocol, GnRH-a antagonist protocol, GnRH-a micro-stimulation protocol, GnRH-a short protocol and antagonist protocol, GnRH-a short protocol and oral contraceptives); (2) study with self-control or other factors that cause infertility, such as severe male factor; (3) repeat publications, case reports, reviews, systematic reviews, and conference papers; (4) documents from which complete data cannot be extracted.

### Data extraction and quality assessment

The data was extracted using Microsoft Excel, and the extracted content included the first author’s name, year of publication, research country, study design type, sample size, ASRM classification, ovarian stimulation program, and outcome index. The risk-of-bias risk assessment tool of the Cochrane Collaboration (version 5.1.0) was used to assess the randomized studies, and the Newcastle-Ottawa Scale (NOS) was used to assess the quality of the included non-RCT studies.

### Outcome indicators

Main indicators: implantation rate. Secondary indicators: (1) fertilization rate; (2) clinical pregnancy rate; (3) basal follicle-stimulating hormone (FSH); (4) duration of ovarian stimulation (days); (5) dose of gonadotropin; (6) number of retrieved oocytes. Supplement  indicators: (1) age; (2) body mass index (BMI); (3) antral follicle count (AFC); (4) basal estradiol hormone (E2); (5) cancer antigen 125 (CA125).

### Statistical analyses

Statistical analyses were performed using Review Manager software (version 5.3; Copenhagen: Nordic Cochrane Centre, Cochrane Collaboration, 2014). The relative risk (RR) was used for the categorical variables, and the standardized mean difference (SMD) was used for the continuous variables as the statistics for the efficacy analysis. All statistics gave a 95% confidence interval (CI). Heterogeneity was assessed by means of the I2 statistic to select fixed-effects (I2 ≤ 50%) or random-effects models (I2 > 50%). When I2 > 50%, it indicated considerable heterogeneity between the studies. The reason was analyzed and the heterogeneity was treated by subgroup analysis or sensitivity analysis and, after homogeneity, the fixed-effects model also could be used. *P* < 0.05 was considered statistically significant. In addition, we did not select the group of patients which benefits from down regulation for a separate analysis, as this might result in selection bias. But we performed subgroup analyses based on study design type to minimize bias.

## Results

### Characteristics of included studies

**Fig. 1:**
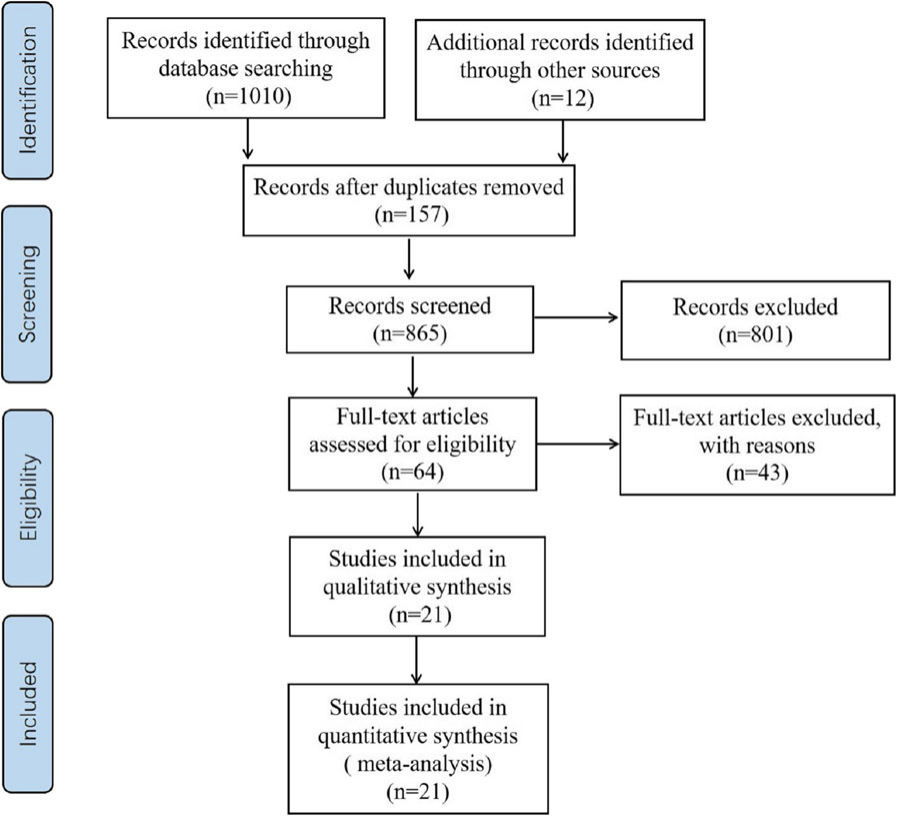
Flowchart showing the selection process for the meta-analysis

The preliminary search yielded 1022 articles. After screening, 21 articles were finally included in the meta-analysis (seven randomized controlled trials and 14 cohort studies) [11–31]. Eight articles were in English and 13 articles were in Chinese. The article search and screening process is shown in Fig. 1. The basic characteristics of the included articles appear in Table 1, Additional file 1: Table S1  and Additional file 4: Table S4.

**Table 1:** Characteristics of the included studies

Included studies	Country	Study type	Protocol (Sample size)			Stage of ASRM
First author /Year			Ultra-long	Long	Short	
Maged 2018 [11]	Egypt	RCT	45	45		Stage III-IV
Decleer 2016 [12]	Belgium	RCT	61	58		Stage I-II
Rickes 2002 [13]	Germany	RCT	45	37		Stage II-IV
Surrey 2002 [14]	American	RCT	25	26		Stage I-IV
Jiang HL 2018 [15]	China	RCT	56	56		Clinical pathology diagnosis
Dai L 2017 [16]	China	RCT	30	30		Laparoscopic or open-abdominal diagnosis
Lin WQ 2004 [17]	China	RCT	26	36		Stage III-IV
Sõritsa 2015 [18]	Estonia	RC	89	53		Stage I-II
Tamura 2014 [19]	Japan	RC	11	12		Stage III-IV
Ma 2008 [20]	China	PC	75	97		Stage III-IV
Nakamura 1992 [21]	Japan	RC	21	11		Stage I-IV
Wang F 2017 [22]	China	RC	102	80		Stage I-II
Du H 2017 [23]	China	RC	42	38	40	Stage III-IV
Jiang YH 2016 [24]	China	RC	30	58	38	Stage III-IV
Zhang QF 2015 [25]	China	RC	25	119	21	Stage I-II
Song N 2014 [26]	China	RC	75	33		Stage III-IV
Deng HL 2012 [27]	China	RC	74	177		Stage I-IV
Sun YL 2012 [28]	China	RC	132	74	56	Stage I-IV
Niu HY 2011 [29]	China	RC	25	25		Stage III-IV
Cheng D 2010 [30]	China	RC	23	67	49	Stage III-IV
Wang L 2009 [31]	China	RC	42	40		Stage III-IV

*PC* prospective cohort, *RC* retrospective cohort, *RCT* randomized controlled trial

### Quality evaluation of included studies

The quality of the 21 studies was evaluated using the Cochrane Collaboration’s bias risk assessment tool and the NOS scale. All seven RCT studies were biased with high risk due to the lack of intentional analysis, and the remaining biases were low risk. The quality evaluation of the RCT studies is shown in Additional file 2: Table S2; the NOS score for the non-RCT study was 7–8, and the quality evaluation is shown in Additional file 3: Table S3.

### Implantation rate

**Fig. 2:**
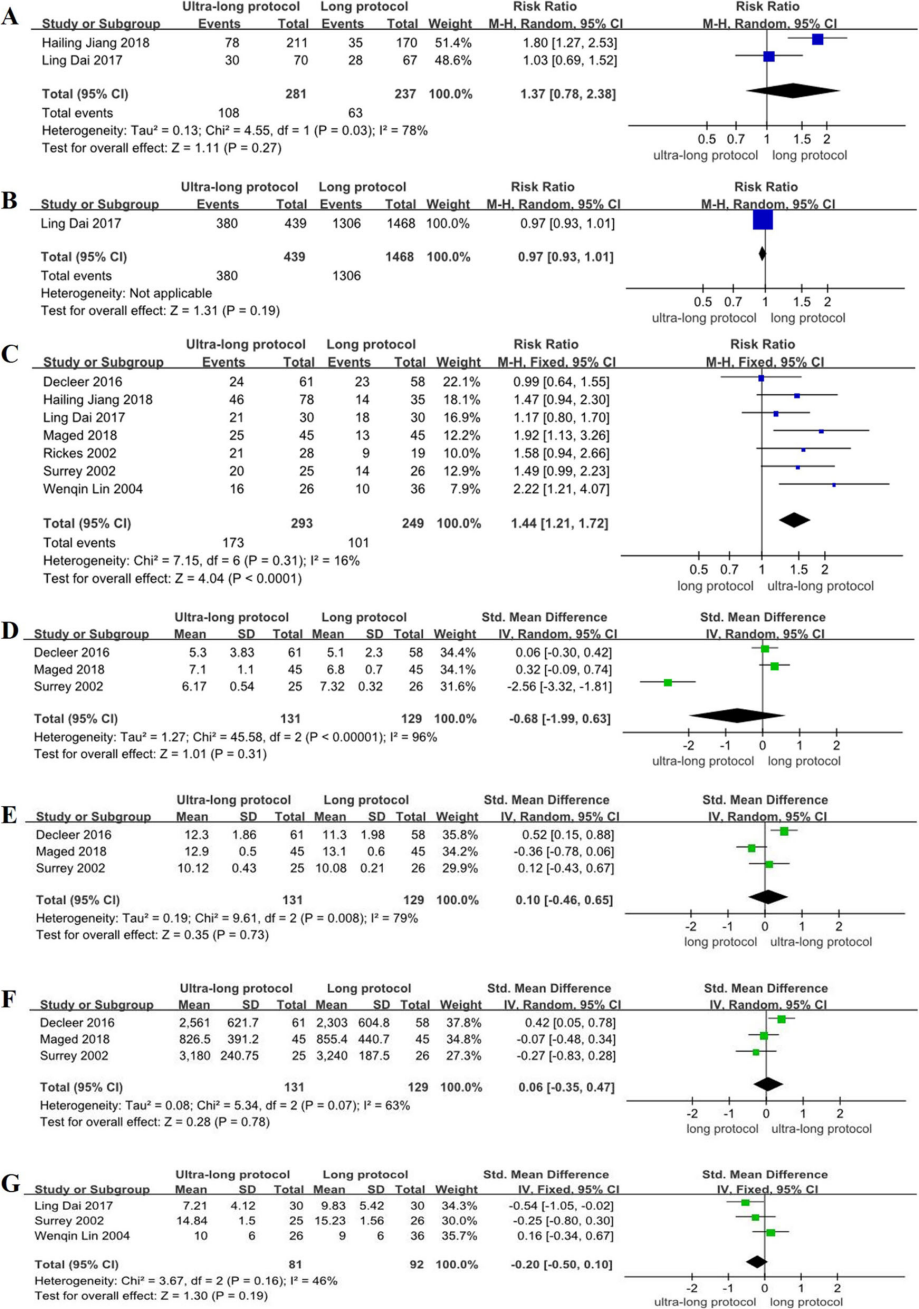
Meta-analysis on implantation rate (**a**), fertilization rate (**b**), clinical pregnancy rate (**c**), basal FSH (**d**), duration of stimulation (**e**), dose of gonadotropin (**f**), the number of retrieved oocytes (**g**): the ultra-long protocol versus long protocol in RCTs

**Fig. 3:**
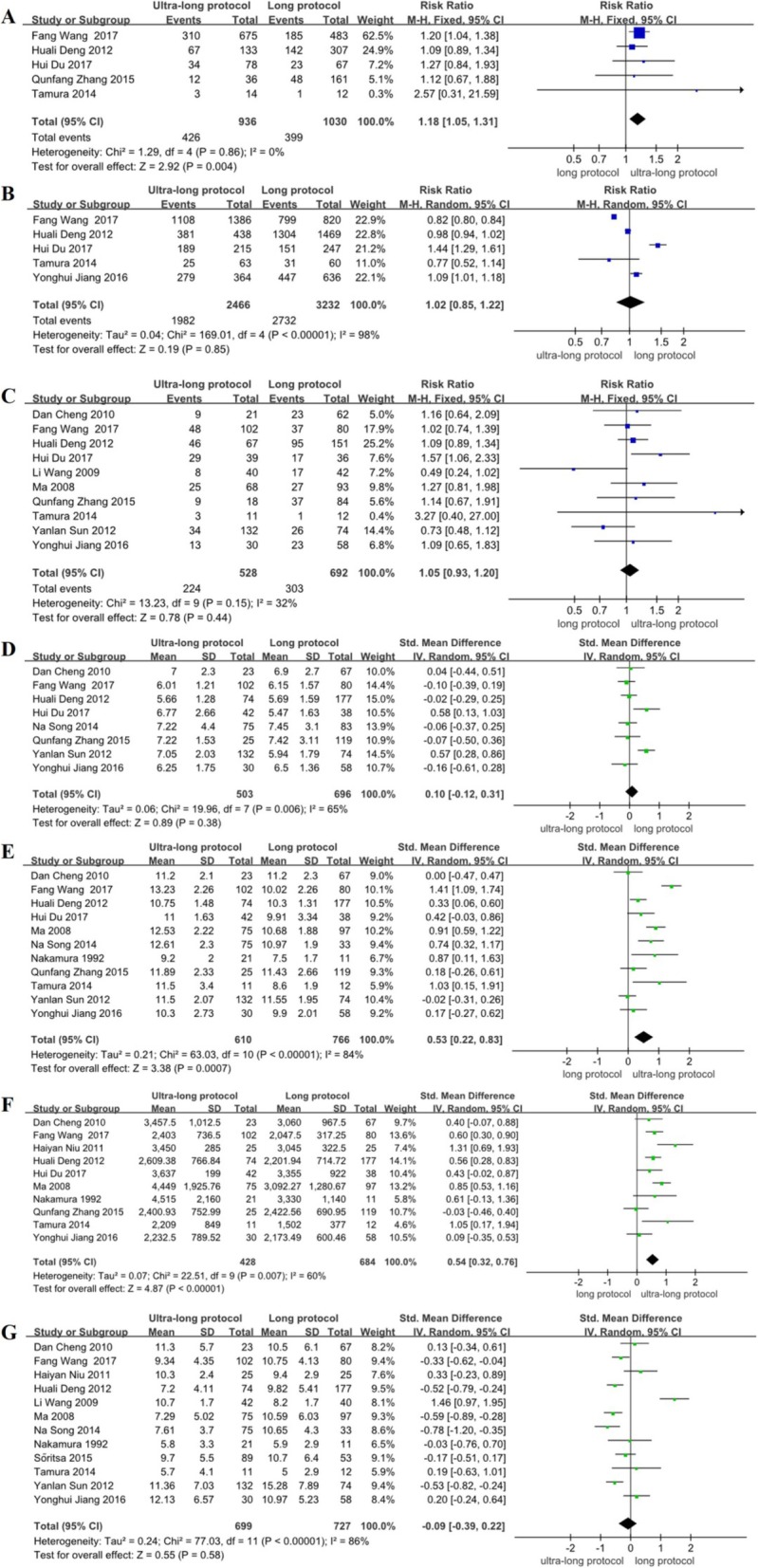
Meta-analysis on implantation rate (**a**), fertilization rate (**b**), clinical pregnancy rate (**c**), basal FSH (**d**), duration of stimulation (**e**), dose of gonadotropin (**f**), the number of retrieved oocytes (**g**): the ultra-long protocol versus long protocol in non-RCTs

**Fig. 4:**
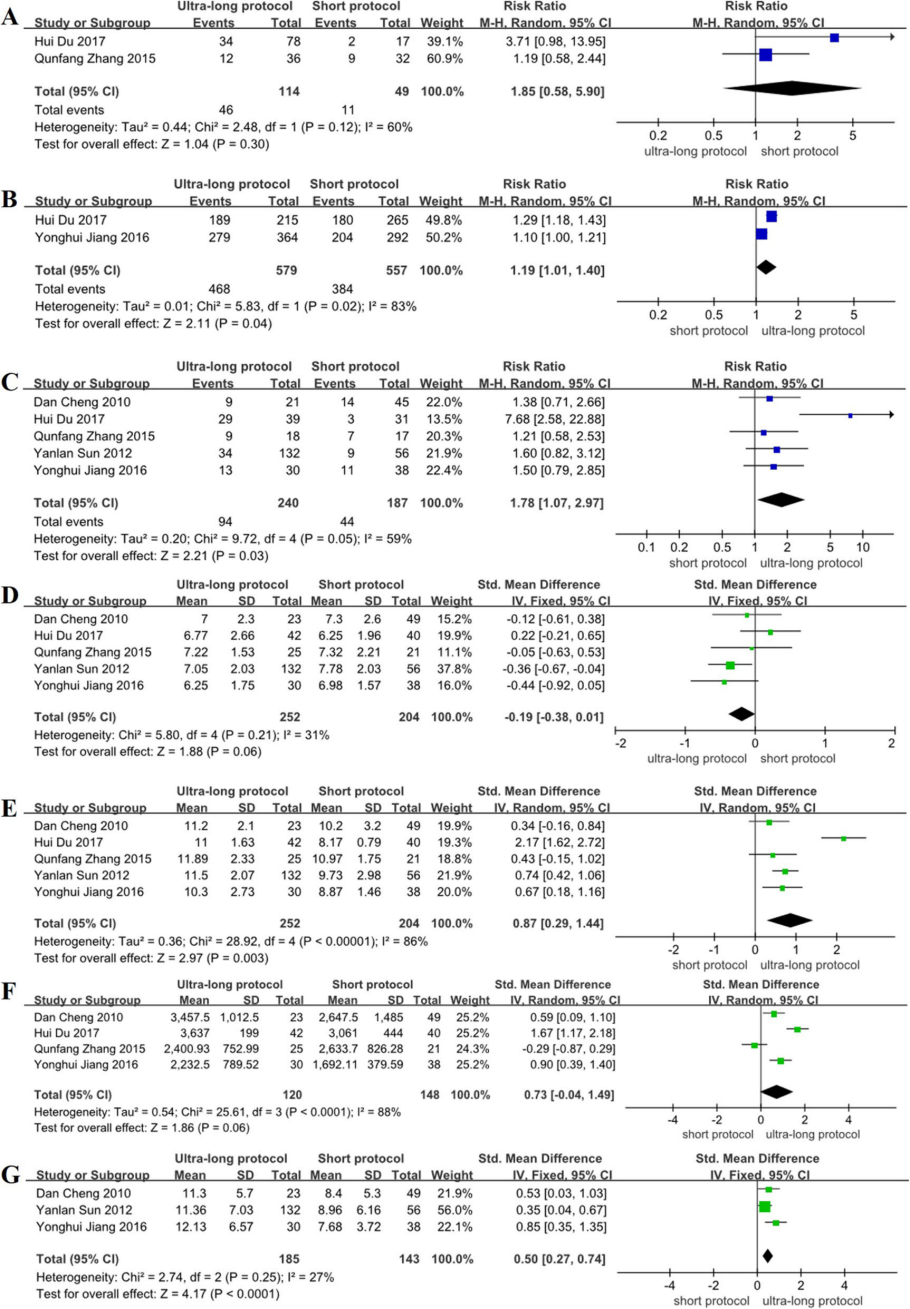
Meta-analysis on implantation rate (**a**), fertilization rate (**b**), clinical pregnancy rate (**c**), basal FSH (**d**), duration of stimulation (**e**), dose of gonadotropin (**f**), the number of retrieved oocytes (**g**): the ultra-long protocol versus short protocol in non-RCTs

Of the subgroup analysis results, the RCT studies found no significant difference about the implantation rate between the ultra-long protocol group and the long protocol group (RR = 1.37, 95% CI: 0.78~2.38, *P* > 0.05) (Fig. 2a); the non-RCT studies found that the implantation rate of the ultra-long protocol group was higher than that of the long protocol group, and the difference was statistically significant (RR = 1.18, 95% CI: 1.05~1.31, *P* < 0.05) (Fig. 3a). Compared with the implantation rate of the ultra-long protocol group and the short protocol group, the non-RCT studies found there was no significant difference between the two groups (RR = 1.85, 95% CI: 0.58~5.90, *P* > 0.05) (Fig. 4a).

### Fertilization rate

The subgroup analysis of the fertilization rate in the ultra-long protocol group and the long protocol group showed that the RCT studies found no significant difference between the two groups (RR = 0.97, 95% CI: 0.93~1.01, *P* > 0.05) (Fig. 2b); there was also no statistical difference in the non-RCT studies between the two groups (RR = 1.02, 95% CI: 0.85~1.22, *P* > 0.05) (Fig. 3b). However, the ultra-long protocol group had a higher fertilization rate than that in the short protocol group in the non-RCT studies, and the difference was statistically significant (RR = 1.19, 95% CI: 1.01~1.40, *P* < 0.05) (Fig. 4b).

### Clinical pregnancy rate

The subgroup analysis results in the RCT studies showed that the clinical pregnancy rate was significantly higher in the ultra-long protocol group than that in the long protocol (RR = 1.44, 95% CI: 1.21~1.72, *P* < 0.05) (Fig. 2c), but no significant difference was found in the non-RCT studies between the two groups (RR = 1.05, 95% CI: 0.93~1.20, *P* > 0.05) (Fig. 3c). In the non-RCT studies, the clinical pregnancy rate of the ultra-long protocol group was higher than it was in the short protocol group (RR = 1.78, 95% CI: 1.07~2.97, *P* < 0.05) (Fig. 4c).

### Basal follicle-stimulating hormone

In subgroup analysis among the RCTs, the ultra-long protocol group (compared with long protocol group) were not associated with any significant differences in the basal FSH (SMD = − 0.68, 95% CI: − 1.99~0.63, *P* > 0.05) (Fig. 2d), and the difference in the basal FSH did not reach statistical significance for these two groups in the non-RCT studies (SMD = 0.10, 95% CI: − 0.12~0.31, *P* > 0.05) (Fig. 3d). Compared with the ultra-long protocol group and the short protocol group, there was also no statistical difference in the basal FSH in the non-RCT studies (SMD = − 0.19, 95% CI: − 0.38~0.01, *P* > 0.05) (Fig. 4d).

### Duration of ovarian stimulation (days)

The subgroup analysis in the RCTs showed that the ultra-long protocol group and the long protocol group did not differ with regard to duration of controlled ovarian hyperstimulation (COH) (SMD = 0.10, 95% CI: − 0.46~0.65, *P* > 0.05) (Fig. 2e); among the non-RCTs, the differences between the two groups were significantly different (higher duration of COH in the ultra-long protocol, SMD = 0.53, 95% CI: 0.22~0.83, *P* < 0.05) (Fig. 3e). The number of stimulation days was higher in the ultra-long protocol group (versus the short protocol group) in the non-RCT studies, and the difference was statistically significant (SMD = 0.87, 95% CI: 0.29~1.44, *P* < 0.05) (Fig. 4e).

### Dose of gonadotropin

Between the ultra-long protocol group and the long protocol group, the subgroup analysis showed that the RCTs found that the dose of gonadotropin did not differ between the two groups (SMD = 0.06, 95% CI: − 0.35~0.47, *P* > 0.05) (Fig. 2f); in the non-RCTs, a statistical difference was found in the dose of gonadotropin, and the ultra-long protocol had a higher dose of gonadotropin than did the long protocol group (SMD = 0.54, 95% CI: 0.32~0.76, *P* < 0.05) (Fig. 3f). Comparison of the dose of gonadotropin in the ultra-long protocol group and the short protocol group showed no difference between the groups in the non-RCTs (SMD = 0.73, 95% CI: − 0.04~1.49, *P* > 0.05) (Fig. 4f).

### Number of retrieved oocytes

Subgroup analysis among the RCTs showed that the number of retrieved oocytes did not differ significantly between the ultra-long protocol group and the long protocol group (SMD = − 0.20, 95% CI: − 0.50~0.10, *P* > 0.05) (Fig. 2g); there was also no statistical difference among the non-RCTs between the two groups (SMD = − 0.09, 95% CI: − 0.39~0.22, *P* > 0.05) (Fig. 3g). Between the ultra-long protocol group and the short protocol group, the number of retrieved oocytes was significantly higher in the ultra-long protocol group in the non-RCTs (SMD = 0.50, 95% CI: 0.27~0.74, *P* < 0.05) (Fig. 4g).

### Comparison of the clinical pregnancy rate among endometriosis patients at stages I–II and stages III–IV

**Fig. 5:**
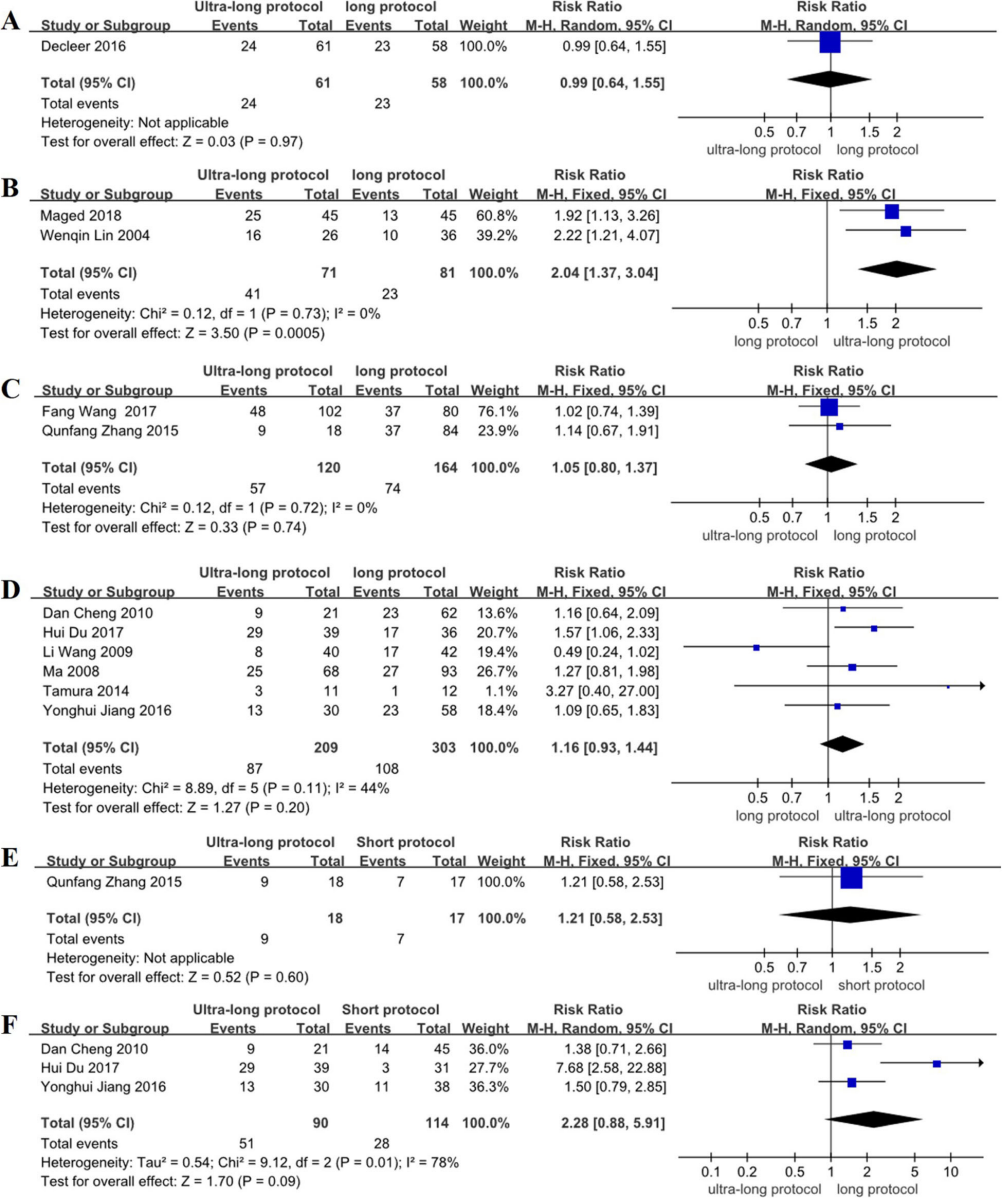
Meta-analysis on clinical pregnancy rate of endometriosis infertility patients at stage I-II(**a**) and stage III-IV(**b**) according to ASRM classification: the ultra-long protocol versus long protocol in RCTs. Clinical pregnancy rate of endometriosis infertility patients at stage I-II(**c**) and stage III-IV(**d**) according to ASRM classification in non-RCTs. Clinical pregnancy rate of endometriosis infertility patients at stage I-II(**e**) and stage III-IV(**f**) according to ASRM classification: the ultra-long protocol and the short protocol in non-RCTs

In subgroup analysis of the RCTs, the ultra-long protocol group (compared with the long protocol group) was not associated with any significant differences in the clinical pregnancy rate of endometriosis patients at stages I–II (RR = 0.99, 95% CI: 0.64~1.55, *P* > 0.05) (Fig. 5a), and no statistically significant difference was found between the two groups in the non-RCT studies (RR = 1.05, 95% CI: 0.80~1.37, *P* > 0.05) (Fig. 5c). Between the ultra-long protocol group and the short protocol group, there was also no difference in the clinical pregnancy rate of endometriosis patients at stages I–II in the non-RCT studies (RR = 1.21, 95% CI: 0.58~2.53, *P* > 0.05) (Fig. 5e).

The subgroup analysis of the RCTs showed that the ultra-long protocol group had a significantly higher clinical pregnancy rate among endometriosis patients at stages III–IV than those in the long protocol group (RR = 2.04, 95% CI: 1.37~3.04, *P* < 0.05) (Fig. 5b), but no statistically significant difference was found between the two groups in the non-RCT studies (RR = 1.16, 95% CI: 0.93~1.44, *P* > 0.05) (Fig. 5d). No difference was found in the clinical pregnancy rate between the ultra-long protocol group and the short protocol group of endometriosis patients at stages III–IV in the non-RCT studies (RR = 2.28, 95% CI: 0.88~5.91, *P* > 0.05) (Fig. 5f).

## Discussion

This study indicated that compared with the GnRH-a long protocol, the GnRH-a ultra-long protocol improved the clinical pregnancy rate or implantation rate of infertile patients, which might improve the clinical outcomes of patients with endometriosis. A previous study reported that GnRH-a pretreatment can improve the microenvironment in the ovary and the quality of oocytes [7]. In addition, it could enhance the expression of endometrial αγβ3 integrin as a hallmark of endometrial receptivity, increase the number of pinopodes that are important for embryo transfer [32], and increase the apoptotic activity of endometrial cells in patients with endometriosis and repair its reduced sensitivity thus increasing its implantation rate [33]. Pretreatment with GnRH-a could also reduce the concentration of interleukin-1, tumor necrosis factor, and nitric oxide that are toxic to embryos in the peritoneal fluid [8, 34] it could make pituitary cells unresponsive to endogenous GnRH-a to achieve desensitization, reduce the secretion of FSH and luteinizing hormone (LH), inhibit ovarian activity, reduce the level of estradiol, and promote atrophy of ectopic foci to favor embryo implantation [35]. However, the dose and time of the drug were extended, and the ultra-long protocol had too deep an inhibition on the pituitary gland, which led to a decrease of ovarian reactivity to gonadotropin. To solve this problem, studies have shown that the level of serum estradiol (E2) after GnRH-a down-regulation was measured to determine whether the patient’s ovaries recovered responsiveness, and gonadotropin was initiated when the E2 value began to rise. Compared with the conventional ultra-long protocol, it could significantly reduce the dosage of gonadotropin used without affecting pregnancy outcomes [36]. This meta-analysis found that among RCTs comparing the GnRH-a long protocol and the GnRH-a ultra-long protocol, the GnRH-a ultra-long protocol mainly improved the clinical pregnancy rate. However, among non-RCTs, the ultra-long protocol mainly improved the implantation rate. The observed differences between the results within RCTs and non-RCTs demonstrate that reliance on neither should be absolute; this difference might be due to analytical methods and confounding factors. Similar to the results of RCTs combining with non-RCTs in this study, a recent Cochrane review (8 RCTs included) indicated that the long-term GnRH agonist therapy had uncertain impacts on the pregnancy outcomes [37]. However, it contradicted with the results of RCTs (7 RCTs included) in this study, which was due to different inclusion criteria and different GnRH-a down-regulation protocols (minimum 3 months versus 1 to 3 months mostly) on infertility patients with endometriosis.

Each of these two types of study designs had strengths and limitations. Although randomized controlled trials could reduce or eliminate the imbalance between the treatment and control groups by random, double-blind, or other methods, RCT studies had strict entry criteria, required greater adherence, lacked universality, and were more expensive. Therefore, the sample sizes of the RCT studies included in this study were small. However, observational studies might be more universal and less affected by selection bias, allowing for larger sample sizes to be collected. As such, both types of data were useful in assessing the effectiveness of various protocols in the treatment of IVF-ET among infertility patients with endometriosis.

The study also further analyzed the clinical pregnancy rate based on the ASRM classification, and the stage I and II were classified according to the ASRM criteria that belonged to a domaine of surgery. The findings were as follows. In RCTs, the GnRH-a ultra-long protocol significantly improved the clinical pregnancy rate among endometriosis patients at stages III–IV compared with the long-term GnRH-a protocol but had no effect on patients with endometriosis at stages I–II. This might be because, compared with patients at stages I–II, changes in pelvic anatomy, the level of inflammatory response in the pelvic cavity, and the degree of the local micro-environment imbalance in the endometrium were all heavier in patients in phases III–IV; therefore, it took a long time and a large dose of down-regulation to inhibit the inflammatory response and improve the pelvic microenvironment. However, the ultra-long protocol also had some potential risks, such as hyper-inhibition of the pituitary and long-term low levels of endogenous FSH, which might lead to fewer numbers of follicles and smaller follicular diameter, which might increase the amount of gonadotropin and reduce the number of retrieved oocytes [38]. For endometriosis patients at stages I–II, the conventional long protocol had been able to achieve the same clinical outcome as the ultra-long protocol; thus, the long protocol might be a cost-effective controlled ovarian hyperstimulation (COH) protocol for patients with stages I–II endometriosis. For patients with severe endometriosis stage III-IV, the GnRH-a ultra-long protocol could achieve better pregnancy outcomes, and this was similar to previous literature reports [23, 39]. Although it has been confirmed by a lot of literatures which suggested that the GnRH-a ultra-long protocol could improve pregnancy outcomes with stages III-IV endometriosis, most of the published RCT or non-RCT studies were performed in a small number of patients. Thus, the systematic meta-analysis of RCT with larger number of patients in this study would be required to obtain more reliable results with higher confidence levels.

In the non-RCTs, two studies (*n* = 150) found that the GnRH-a ultra-long protocol improved the fertilization rate compared to the GnRH-a short protocol, which was similar to the results in previous literature [23]. Analysis of three other studies, including 328 patients, showed that the ultra-long protocol could increase the number of retrieved oocytes. In addition, five studies indicated that the ultra-long protocol could also increase the clinical pregnancy rate, but the numbers of stimulation days were longer. Because the five included studies were retrospective cohort studies with smaller sample sizes, this research conclusion only represented current evidence, and the reliability of the result requires prospective and randomized studies with a larger sample size.

The GnRH-a ultra-long protocol, compared to the long protocol, and the results of the RCT studies and the non-RCT studies were all consistent in terms of fertilization rate, basal FSH, and the number of retrieved oocytes, none of which were statistically significant. The ultra-long and the long protocol had no effects on these three aspects. In this study, female age (Additional file 5: Figure S1), BMI (Additional file 6: Figure S2), antral follicle count (AFC) (Additional file 7: Figure S3), basal E2 levels (Additional file 8: Figure S4), and basal LH levels (Additional file 9: Figure S5) were also not significantly different between the GnRH-a ultra-long protocol and the GnRH-a long/short protocol in RCTs and/or non-RCTs. Serum CA125 level was correlated with the severity of endometriosis and treatment effect. In this data, there was statistically significant difference in CA125 levels before down-regulation between the GnRH-a ultra-long protocol and the GnRH-a long protocol in non-RCTs. However, there was no significant difference between the two groups after down-regulation (P >0.05) (Additional file 10: Figure S6). This result might be due to the small sample size and the large inter-group heterogeneity.

Nevertheless, our meta-analysis still had several shortcomings. Even if strict inclusion and exclusion criteria were used, the conclusions drawn from this data were still subject to the limitations of the original studies themselves. Only seven RCTs were included in our meta-analysis, three of which were low-quality due to unclear methods of randomized allocation, and the remaining 14 articles were cohort studies. Although we performed subgroup analyses based on different types of research, heterogeneity was still quite apparent between studies because of the GnRH-a dose, the treatment time, and the starting time of gonadotropin, which were not completely the same in each protocol. Therefore, large-sample, multicenter, randomized controlled trials are still needed to obtain more reliable results.

## Conclusions

This systematic meta-analysis firstly reports the GnRH-a ultra-long protocol can improve the clinical pregnancy rate of infertile patients in RCT studies, especially in patients with stages III–IV endometriosis. However, the results of subgroup analysis suggest that different down-regulation protocols provide no significant differences in improving clinical outcomes in patients with endometriosis in the non-RCT studies. Although it is generally believed that the results of RCT are more reliable, the conclusions of non-RCT studies cannot be easily neglect, which let us drawn conclusions more cautious. Furthermore, the drugs produced by different manufacturers, dose of gonadotropin, duration of ovarian stimulation, starting time of gonadotropin and the ethnic groups were not completely the same in each study. The non-RCT studies had defects in the control of several confounding factors mentioned above, which might lead to bias, and these factors might be precisely the key to affecting the conclusions.

## Supplementary information

**Additional file 1: Table S1.** Characteristics of the included studies.

**Additional file 2: Table S2.** Risk of bias of included RCTs using the Cochrane risk assessment tool.

**Additional file 3: Table S3.** Quality assessment of included cohort studies using the Newcastle–Ottawa Scale.

**Additional file 4: Table S4.** Basic characteristics of the included studies.

**Additional file 5: Figure S1.** Meta-analysis on age: the ultra-long protocol versus long protocol in RCTs (A) and non-RCTs (B).

**Additional file 6: Figure S2.** Meta-analysis on BMI in non-RCTs: the ultra-long protocol versus long protocol (A), and the ultra-long protocol versus short protocol (B).

**Additional file 7: Figure S3.** Meta-analysis on antral follicle count in non-RCTs: the ultra-long protocol versus long protocol (A), and the ultra-long protocol versus short protocol (B).

**Additional file 8: Figure S4.** Meta-analysis on basal E2 levels: the ultra-long protocol versus long protocol in RCTs (A) and in non-RCTs (B), and the ultra-long protocol versus short protocol in non-RCTs (C).

**Additional file 9: Figure S5.** Meta-analysis on basal LH levels in non-RCTs: the ultra-long protocol versus long protocol (A), and the ultra-long protocol versus short protocol (B).

**Additional file 10: Figure S6.** Meta-analysis on CA125 in non-RCTs: the ultra-long protocol versus long protocol before down-regulation (A), and after down-regulation (B).

## Data Availability

Please contact author for data requests.

## Abbreviations

AFC: Antral follicle count
ART: Assisted reproductive technology
ASRM: The American Society for Reproductive Medicine
BMI: Body mass index
CA125: Cancer antigen 125
CNKI: China National Knowledge Infrastructure
COH: Controlled ovarian hyperstimulation
COS: Controlled ovarian stimulation
E2: Estradiol
FSH: Basal follicle-stimulating hormone
ICSI: Intracytoplasmic sperm injection
IVF-ET: In vitro fertilization-embryo transfer
LH: Luteinizing hormone
non-RCTs: Observational studies
NOS: The Newcastle-Ottawa Scale
PC: Prospective cohort
RC: Retrospective cohort
RCT: Randomized controlled trial
RR: The relative risk
SMD: The standardized mean difference
