# Supplementary material for: The effectiveness of different down-regulating protocols on in vitro fertilization-embryo transfer in endometriosis: a meta-analysis
Source: Reprod Biol Endocrinol. 2020 Feb 29;18:16. doi: 10.1186/s12958-020-00571-6 (PMC7049222; doi:10.1186/s12958-020-00571-6)
Supplement: Supplementary file 1 — Additional file 1: Table S1. Characteristics of the included studies. [file 12958_2020_571_MOESM1_ESM.pdf]

Additional file 1: Table S1 Characteristics of the included studies.

Study	Ultra-long protocol	Long protocol	Short protocol	Outcome indicators
Maged [11]	Decapetyl 3.75mg, 3 cycles	Decapetyl 0.1mg		②③④⑦
Decleer [12]	Goserelin 3.6 mg, 3 cycles	Buserelin 900μg		②③④⑦
Rickes [13]	Goserelin 3.6mg, 5-6 cycles	Decapetyl 0.1mg		⑦
Surrey [14]	Leuprorelin 3.75mg, 3 cycles	Leuprorelin 0.5~1.0mg		①②③④⑤⑥⑦
Jiang HL [15]	3.75mg, 3 cycles, Unknown drug	Unknown 0.1mg		①⑦
Dai L [16]	3.75mg, 3 cycles, Unknown drug	Triptorelin 0.1mg		①⑤⑥⑦
Lin WQ [17]	Goserelin 3.6mg, 3 cycles	Decapetyl 0.1mg		①⑤⑦
Sõritsa [18]	Diphereline 3.75mg, 3 cycles	No mention		⑤
Tamura [19]	Buserelin 1.8 mg, 3 cycles	Buserelin 900μg		①③④⑤⑥⑦
Ma [20]	Diphereline 3.75mg, 3 cycles	Diphereline 0.05~0.1mg		①③④⑤⑦
Nakamura [21]	Buserelin 900μg~1200μg, 3 cycles	Buserelin 900μg		③④⑤⑦
Wang F [22]	Leuprorelin 3.75mg, 2 cycles	Decapetyl 0.1mg		①②③④⑤⑥⑦
Du H [23]	Diphereline 3.75mg, 3 cycles	Diphereline 1.25mg	Diphereline 0.1mg	①②③④⑤⑥⑦
Jiang YH [24]	Leuprorelin 3.75mg	Triptorelin 0.1mg	Triptorelin 0.1mg	②③④⑤⑥⑦
Zhang QF [25]	Diphereline 1.25~1.88mg, 2 cycles	Diphereline 1.25~1.88mg	Diphereline 0.05mg	①②③④⑥⑦
Song N [26]	Diphereline 3.75mg, 2-3 cycles	Diphereline 0.1mg		①②③④⑤⑦
Deng HL [27]	Diphereline 3.75mg, 3 cycles	Triptorelin 0.1mg		①②③④⑤⑥⑦
Sun YL [28]	Decapetyl 3.75mg, 2-3 cycles	Decapetyl 1.90mg	Decapetyl 1.33 mg	②③④⑤⑦
Niu HY [29]	Diphereline 3.75mg, 2-3 cycles	Triptorelin 0.1mg		①③⑤⑥⑦
Cheng D [30]	Decapetyl 3.75mg, 2-3 cycles	Triptorelin 0.1mg	Triptorelin 0.1mg	①②③④⑤⑥⑦
Wang L [31]	Decapetyl 3.75mg, 3 cycles	Decapetyl 3.75mg		①⑤⑥⑦

①implantation rate; ②basal FSH level; ③dose of gonadotropin; ④duration of ovarian stimulation(days); ⑤the number of retrieved oocytes; ⑥fertilization rate; ⑦clinical pregnancy rate. One cycle was equivalent to 28 days.
