# Supplementary material for: The effectiveness of different down-regulating protocols on in vitro fertilization-embryo transfer in endometriosis: a meta-analysis
Source: Reprod Biol Endocrinol. 2020 Feb 29;18:16. doi: 10.1186/s12958-020-00571-6 (PMC7049222; doi:10.1186/s12958-020-00571-6)
Supplement: Supplementary file 2 — Additional file 2: Table S2. Risk of bias of included RCTs using the Cochrane risk assessment tool. [file 12958_2020_571_MOESM2_ESM.pdf]

Additional file 2: Table S2 Risk of bias of included RCTs using the Cochrane risk assessment tool.

Included studies	Random sequence generation	Allocation concealment	Blind method		Incomplete outcome data	Selective reporting	Other bias
First author /Year			Blinding of participants and personnel	Blinding of outcome assessment			
Maged 2018[11]	Low risk	Low risk	Low risk	Low risk	Low risk	Low risk	High risk
Decleer 2016[12]	Low risk	Low risk	Low risk	Low risk	Low risk	Low risk	High risk
Rickes 2002[13]	Low risk	Low risk	Low risk	Low risk	Low risk	Low risk	High risk
Surrey 2002[14]	Low risk	Unclear risk	Low risk	Low risk	Low risk	Low risk	High risk
Jiang HL2018[15]	Unclear risk	Unclear risk	Low risk	Low risk	Low risk	Low risk	High risk
Dai L 2017[16]	Unclear risk	Unclear risk	Low risk	Low risk	Low risk	Low risk	High risk
Lin WQ 2004[17]	High risk	Unclear risk	Low risk	Low risk	Low risk	Low risk	High risk
