# Supplementary material for: The effectiveness of different down-regulating protocols on in vitro fertilization-embryo transfer in endometriosis: a meta-analysis
Source: Reprod Biol Endocrinol. 2020 Feb 29;18:16. doi: 10.1186/s12958-020-00571-6 (PMC7049222; doi:10.1186/s12958-020-00571-6)
Supplement: Supplementary file 3 — Additional file 3: Table S3. Quality assessment of included cohort studies using the Newcastle–Ottawa Scale. [file 12958_2020_571_MOESM3_ESM.pdf]

Additional file 3: Table S3 Quality assessment of included cohort studies using the Newcastle–Ottawa Scale.

Included studies	Subject selection				Comparability	Outcome			Score
First author /Year	Representativeness of the exposed cohort	Selection of the non-exposed cohort	Ascertainment of exposure	Demonstration that outcome of interest was not present at start of study	Control of confounding factors	Assessment of outcome	Was follow-up long enough	Adequacy of follow up of cohorts	
Sõritsa 2015[18]	1	1	1		1	1	1	1	7
Tamura 2014[19]	1	1	1		1	1	1	1	7
Ma 2008[20]	1	1	1	1	1	1	1	1	8
Nakamura 1992[21]	1	1	1		1	1	1	1	7
Wang F 2017[22]	1	1	1		1	1	1	1	7
Du H 2017[23]	1	1	1		1	1	1	1	7
Jiang YH 2016[24]	1	1	1		1	1	1	1	7
Zhang QF 2015[25]	1	1	1		1	1	1	1	7
Song N 2014[26]	1	1	1		1	1	1	1	7
Deng HL 2012[27]	1	1	1		1	1	1	1	7
Sun YL 2012[28]	1	1	1		1	1	1	1	7
Niu HY 2011[29]	1	1	1		1	1	1	1	7
Cheng D 2010[30]	1	1	1		1	1	1	1	7
Wang L 2009[31]	1	1	1		1	1	1	1	7
