# Supplementary material for: The effectiveness of different down-regulating protocols on in vitro fertilization-embryo transfer in endometriosis: a meta-analysis
Source: Reprod Biol Endocrinol. 2020 Feb 29;18:16. doi: 10.1186/s12958-020-00571-6 (PMC7049222; doi:10.1186/s12958-020-00571-6)
Supplement: Supplementary file 4 — Additional file 4: Table S4. Basic characteristics of the included studies. [file 12958_2020_571_MOESM4_ESM.pdf]

Additional file 4: Table S4 Basic characteristics of the included studies.

Included studies<br>First author /Year	Protocol	Age (years)	BMI (kg/m^2)	AFC (n)	Basal E2 Levels (pg/ml)	Basal LH levels (IU/L)	CA125 before down-regulation (KU/L)	CA125 after down-regulation (KU/L)	Medical treatments before IVF cycle
Maged 2018[11]	Ultra-long	31.20±1.40			53.10±3.70	6.20±0.90			Without hormone therapy for 1 years
	Long	30.80±1.20			51.90±3.50	5.90±0.80			
Decleer 2016[12]	Ultra-long	30.30±3.63	23.40±4.20						Surgical treatment
	Long	31.70±4.28	23.10±4.58						
Rickes 2002[13]	Ultra-long								Surgical treatment
	Long								
Surrey 2002[14]	Ultra-long	33.12±0.67			30.26±5.37				Surgical treatment
	Long	32.58±0.56			30.23±4.84				
Jiang HL 2018[15]	Ultra-long	30.95±5.37							No mentioned
	Long	31.74±5.28							
Dai L 2017[16]	Ultra-long								No mentioned
	Long								
Lin WQ 2004[17]	Ultra-long	31.00±4.00							Without hormone therapy for 3 months
	Long	32.00±3.00							
Sõritsa 2015[18]	Ultra-long	33.30±4.70							No mentioned
	Long	33.00±3.90							
Tamura 2014[19]	Ultra-long	33.50±3.30							Without hormone therapy
	Long	34.50±3.40							
Ma 2008[20]	Ultra-long	32.91±3.41							No mentioned
	Long	32.91±3.02							
Nakamura 1992[21]	Ultra-long	31.40±3.20							No mentioned
	Long	33.60±2.60							
Wang F 2017[22]	Ultra-long	30.67±4.52	21.89±2.80	10.92±2.73	40.11±3.86		39.59±12.16	35.57±10.17	No mentioned
	Long	31.27±2.52	22.45±1.73	9.56±2.85	42.23±2.76		31.03±20.14	31.93±17.29	
Du H 2017[23]	Ultra-long	32.69±3.62	21.93±1.60	4.98±1.95	40.57±19.58	5.74±8.41			Without hormone therapy and immunosuppressant for 3 months
	Long	31.54±2.11	21.51±1.59	5.16±1.48	50.69±35.29	3.23±0.97			
	Short	33.45±2.79	22.48±1.32	5.38±1.22	53.34±29.85	4.42±1.71			

Continued.

Jiang YH 2016[24]	Ultra-long	30.63±3.60	22.59±3.16		39.48±12.07				No mentioned
	Long	30.24±4.09	22.57±3.11		38.95±12.87				
	Short	31.61±3.41	22.47±2.25		38.90±11.47				
Zhang QF 2015[25]	Ultra-long	30.37±3.53	20.25±2.14		56.33±4.52	4.71±2.53			Without hormone therapy and immunosuppressant for 6 months
	Long	31.44±4.36	21.39±5.75		53.74±38.16	5.51±2.00			
	Short	30.36±3.37	21.98±4.56		54.41±35.68	4.80±2.23			
Song N 2014[26]	Ultra-long	33.60±4.50			48.70±38.30	5.28±5.90			No mentioned
	Long	32.90±4.10			63.50±28.80	4.38±1.90			
Deng HL 2012[27]	Ultra-long	30.84±2.67	21.24±1.86		33.00±14.61	3.86±2.81	53.25±41.57	15.89±7.18	No mentioned
	Long	30.49±3.11	21.06±2.09		31.73±14.98	3.32±1.19	21.43±12.74	18.75±8.21	
Sun YL 2012[28]	Ultra-long	31.37±3.44		8.27±2.75	57.18±31.58	4.77±2.52			No mentioned
	Long	31.82±3.04		10.34±2.61	56.53±29.98	4.54±2.12			
	Short	33.14±3.92		4.30±1.73	48.52±30.53	4.93±3.12			
Niu HY 2011[29]	Ultra-long	31.50±2.50		10.20±3.10	45.20±8.70				No mentioned
	Long	32.10±2.70		10.50±3.40	46.10±7.90				
Cheng D 2010[30]	Ultra-long	31.70±4.10		10.30±4.50	49.80±12.60				No mentioned
	Long	31.30±3.50		10.70±5.10	56.80±14.80				
	Short	33.10±3.10		9.90±4.30	54.30±13.30				
Wang L 2009[31]	Ultra-long	32.10±3.10							No mentioned
	Long	31.70±2.30							

AFC: Antral follicle count.
