# Supplementary figures and images for: The effectiveness of different down-regulating protocols on in vitro fertilization-embryo transfer in endometriosis: a meta-analysis
Source: Reprod Biol Endocrinol. 2020 Feb 29;18:16. doi: 10.1186/s12958-020-00571-6 (PMC7049222; doi:10.1186/s12958-020-00571-6)

A

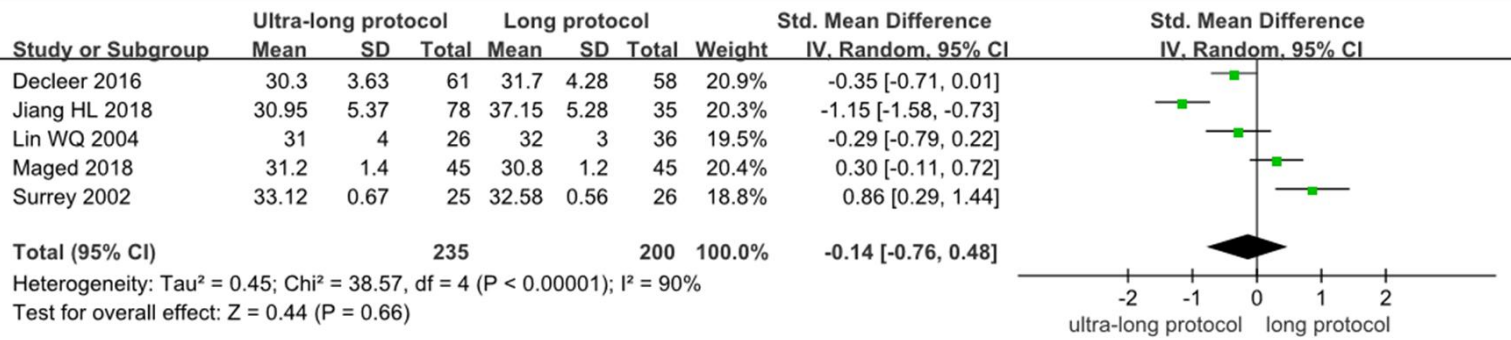

B

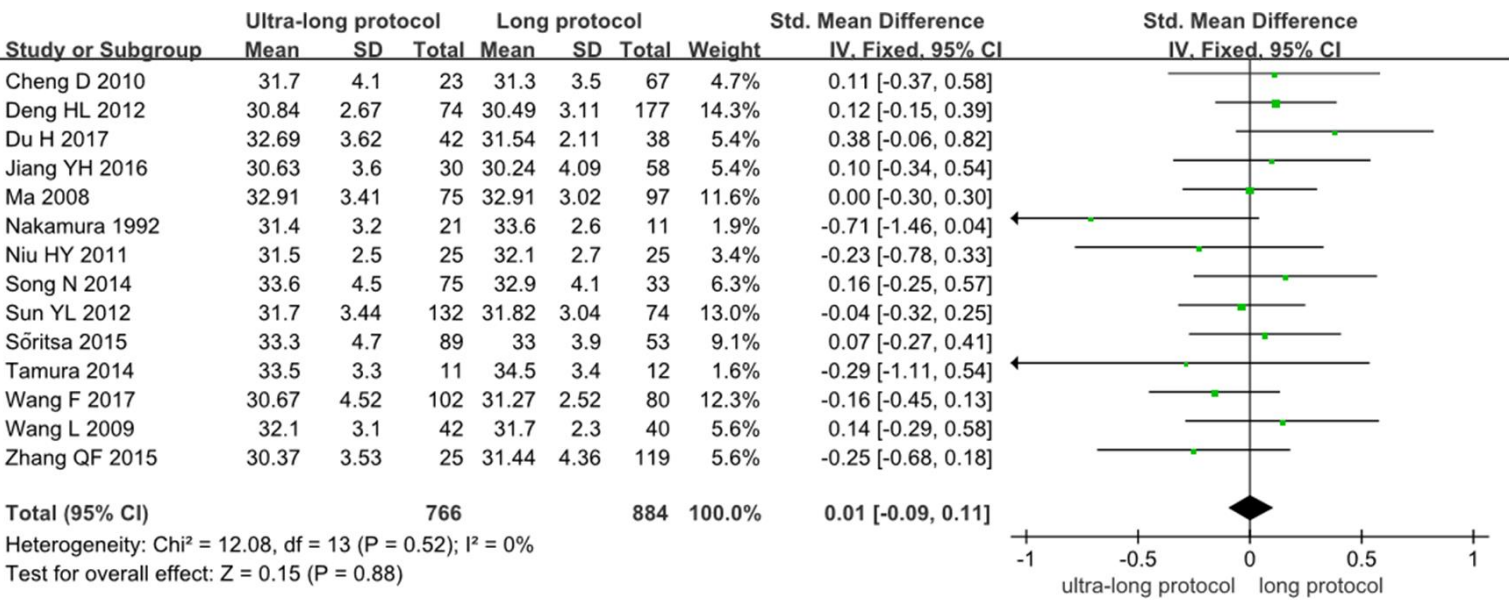

**Fig. S1** Meta-analysis on age: the ultra-long protocol versus long protocol in RCTs (A) and non-RCTs (B).

Supplement: Supplementary file 5 — Additional file 5: Figure S1. Meta-analysis on age: the ultra-long protocol versus long protocol in RCTs (A) and non-RCTs (B). [file 12958_2020_571_MOESM5_ESM.pdf]
