# Supplementary material for: The effectiveness of different down-regulating protocols on in vitro fertilization-embryo transfer in endometriosis: a meta-analysis
Source: Reprod Biol Endocrinol. 2020 Feb 29;18:16. doi: 10.1186/s12958-020-00571-6 (PMC7049222; doi:10.1186/s12958-020-00571-6)
Supplement: Supplementary file 6 — Additional file 6: Figure S2. Meta-analysis on BMI in non-RCTs: the ultra-long protocol versus long protocol (A), and the ultra-long protocol versus short protocol (B). [file 12958_2020_571_MOESM6_ESM.pdf]

**A**

| Study or Subgroup | Ultra-long protocol Mean | SD | Total | Long protocol Mean | SD | Total | Weight | Std. Mean Difference IV, Fixed, 95% CI |
|---|---|---|---|---|---|---|---|---|
| Deng HL 2012 | 21.24 | 1.86 | 74 | 21.06 | 2.09 | 177 | 33.3% | 0.09 [-0.18, 0.36] |
| Du H 2017 | 21.93 | 1.6 | 42 | 21.51 | 1.59 | 38 | 12.6% | 0.26 [-0.18, 0.70] |
| Jiang YH 2016 | 22.59 | 3.16 | 30 | 22.57 | 3.11 | 58 | 12.6% | 0.01 [-0.43, 0.45] |
| Wang F 2017 | 21.89 | 2.8 | 102 | 22.45 | 1.73 | 80 | 28.4% | -0.23 [-0.53, 0.06] |
| Zhang QF 2015 | 20.25 | 2.14 | 25 | 21.39 | 5.75 | 119 | 13.1% | -0.21 [-0.65, 0.22] |
| **Total (95% CI)** | | | **273** | | | **472** | **100.0%** | **-0.03 [-0.19, 0.13]** |

Heterogeneity: Chi² = 4.96, df = 4 (P = 0.29); I² = 19%

Test for overall effect: Z = 0.39 (P = 0.70)

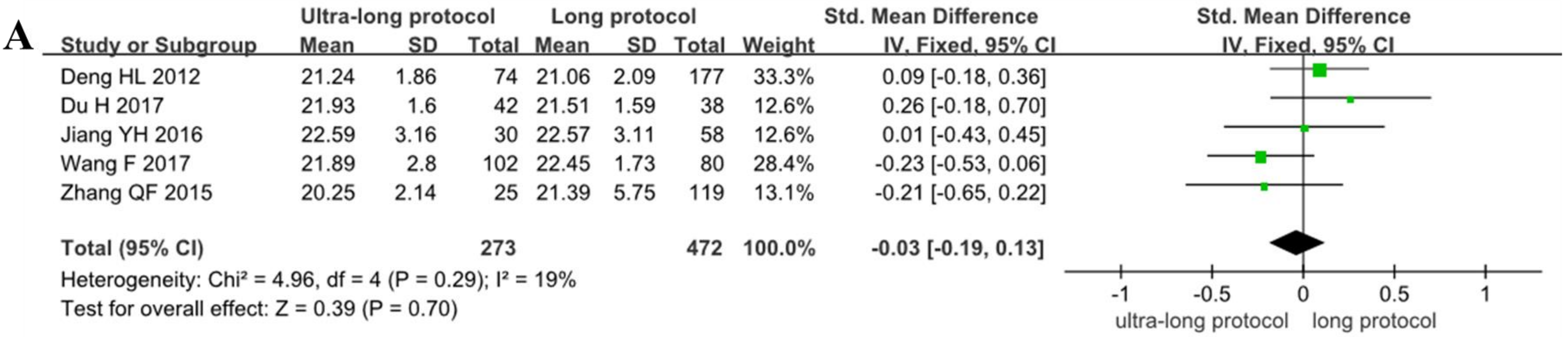


**B**

| Study or Subgroup | Ultra-long protocol Mean | SD | Total | Short protocol Mean | SD | Total | Weight | Std. Mean Difference IV, Fixed, 95% CI |
|---|---|---|---|---|---|---|---|---|
| Du H 2017 | 21.93 | 1.6 | 42 | 22.48 | 1.32 | 40 | 42.0% | -0.37 [-0.81, 0.07] |
| Jiang YH 2016 | 22.59 | 3.16 | 30 | 22.47 | 2.25 | 38 | 35.0% | 0.04 [-0.43, 0.52] |
| Zhang QF 2015 | 20.25 | 2.14 | 25 | 21.98 | 4.56 | 21 | 23.1% | -0.49 [-1.08, 0.10] |
| **Total (95% CI)** | | | **97** | | | **99** | **100.0%** | **-0.25 [-0.54, 0.03]** |

Heterogeneity: Chi² = 2.39, df = 2 (P = 0.30); I² = 16%

Test for overall effect: Z = 1.76 (P = 0.08)

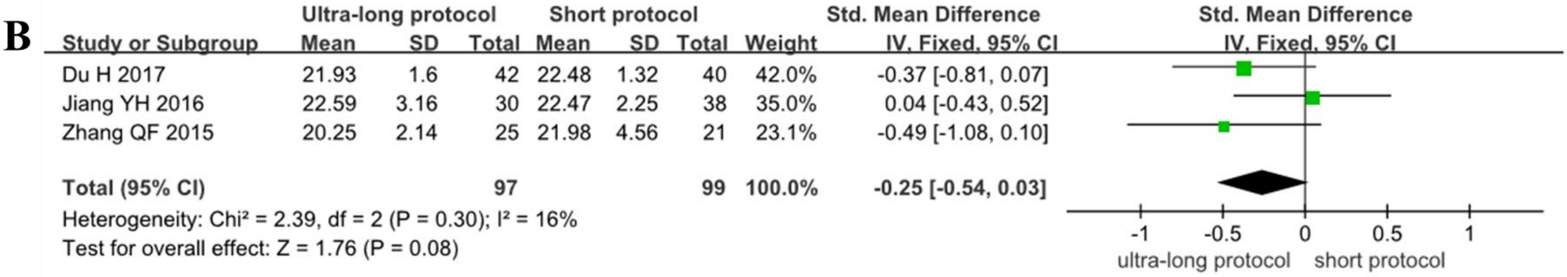


**Fig. S2** Meta-analysis on BMI in non-RCTs: the ultra-long protocol versus long protocol (A), and the ultra-long protocol versus short protocol (B).
