# Supplementary material for: The effectiveness of different down-regulating protocols on in vitro fertilization-embryo transfer in endometriosis: a meta-analysis
Source: Reprod Biol Endocrinol. 2020 Feb 29;18:16. doi: 10.1186/s12958-020-00571-6 (PMC7049222; doi:10.1186/s12958-020-00571-6)
Supplement: Supplementary file 7 — Additional file 7: Figure S3. Meta-analysis on antral follicle count in non-RCTs: the ultra-long protocol versus long protocol (A), and the ultra-long protocol versus short protocol (B). [file 12958_2020_571_MOESM7_ESM.pdf]

A

Study or Subgroup	Ultra-long protocol Mean	SD	Total	Long protocol Mean	SD	Total	Weight	Std. Mean Difference IV, Random, 95% CI
Cheng D 2010	10.3	4.5	23	10.7	5.1	67	19.3%	-0.08 [-0.55, 0.39]
Du H 2017	4.98	1.95	42	5.16	1.48	38	19.8%	-0.10 [-0.54, 0.34]
Niu HY 2011	10.2	3.1	25	10.5	3.4	25	18.3%	-0.09 [-0.65, 0.46]
Sun YL 2012	8.27	2.75	132	10.34	2	74	21.3%	-0.82 [-1.12, -0.53]
Wang F 2017	10.92	2.73	102	9.56	2.85	80	21.3%	0.49 [0.19, 0.78]
**Total (95% CI)**			**324**			**284**	**100.0%**	**-0.12 [-0.66, 0.41]**

Heterogeneity: Tau² = 0.33; Chi² = 37.67, df = 4 (P < 0.00001); I² = 89%
Test for overall effect: Z = 0.45 (P = 0.65)

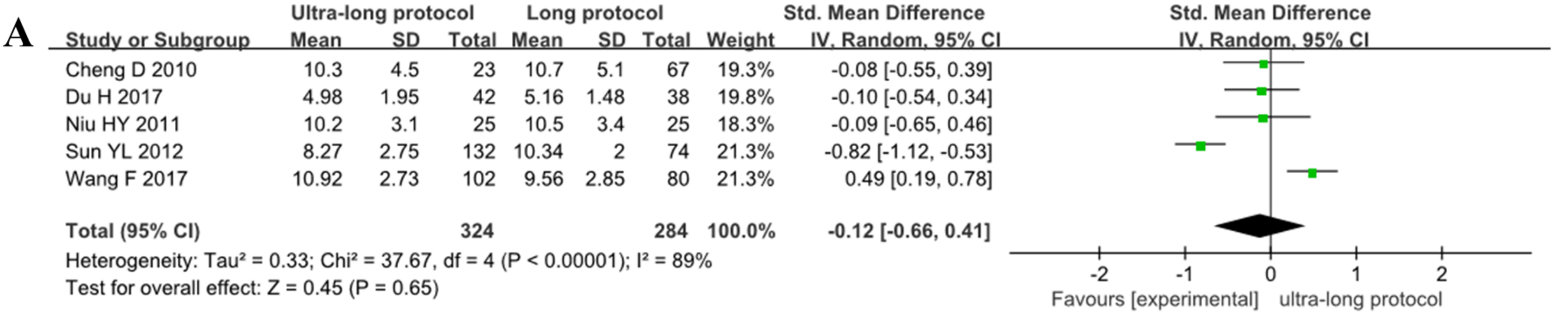

B

Study or Subgroup	Ultra-long protocol Mean	SD	Total	Short protocol Mean	SD	Total	Weight	Std. Mean Difference IV, Random, 95% CI
Cheng D 2010	10.3	4.5	23	9.9	4.3	49	32.9%	0.09 [-0.40, 0.59]
Du H 2017	4.98	1.95	42	5.38	1.22	40	33.3%	-0.24 [-0.68, 0.19]
Sun YL 2012	8.27	2.75	132	4.3	1.73	56	33.8%	1.59 [1.23, 1.94]
**Total (95% CI)**			**197**			**145**	**100.0%**	**0.49 [-0.72, 1.69]**

Heterogeneity: Tau² = 1.08; Chi² = 48.19, df = 2 (P < 0.00001); I² = 96%
Test for overall effect: Z = 0.79 (P = 0.43)

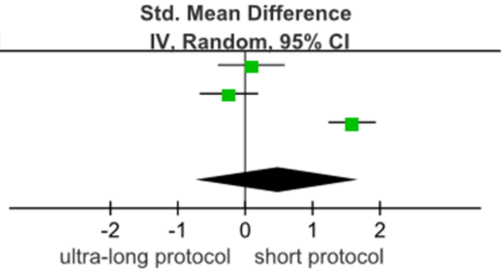

Fig. S3 Meta-analysis on antral follicle count in non-RCTs: the ultra-long protocol versus long protocol (A), and the ultra-long protocol versus short protocol (B).
