# Supplementary material for: The effectiveness of different down-regulating protocols on in vitro fertilization-embryo transfer in endometriosis: a meta-analysis
Source: Reprod Biol Endocrinol. 2020 Feb 29;18:16. doi: 10.1186/s12958-020-00571-6 (PMC7049222; doi:10.1186/s12958-020-00571-6)
Supplement: Supplementary file 8 — Additional file 8: Figure S4. Meta-analysis on basal E2 levels: the ultra-long protocol versus long protocol in RCTs (A) and in non-RCTs (B), and the ultra-long protocol versus short protocol in non-RCTs (C). [file 12958_2020_571_MOESM8_ESM.pdf]

A

Study or Subgroup	Ultra-long protocol Mean	SD	Total	Long protocol Mean	SD	Total	Weight	Std. Mean Difference IV, Fixed, 95% CI
Maged 2018	53.1	3.7	45	51.9	3.5	45	63.5%	0.33 [-0.09, 0.75]
Surrey 2002	30.26	5.37	25	30.23	4.84	26	36.5%	0.01 [-0.54, 0.55]
**Total (95% CI)**			**70**			**71**	**100.0%**	**0.21 [-0.12, 0.54]**

Heterogeneity: Chi² = 0.85, df = 1 (P = 0.36); I² = 0%

Test for overall effect: Z = 1.25 (P = 0.21)

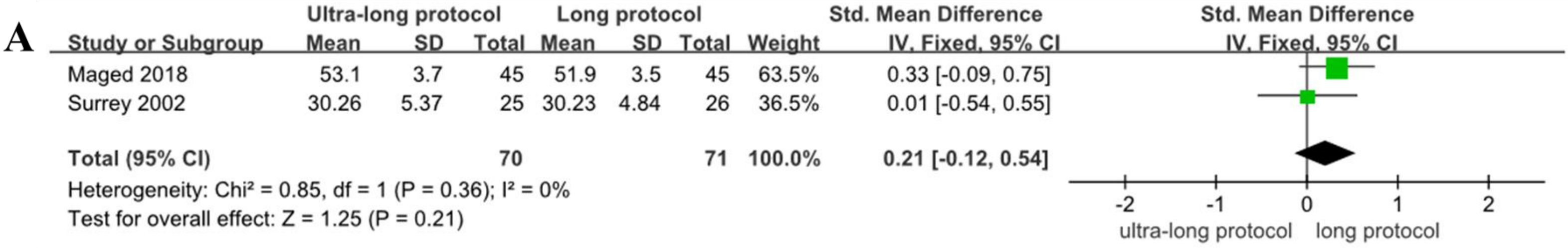

B

Study or Subgroup	Ultra-long protocol Mean	SD	Total	Long protocol Mean	SD	Total	Weight	Std. Mean Difference IV, Random, 95% CI
Cheng D 2010	49.8	12.6	23	56.8	14.8	67	9.2%	-0.49 [-0.97, -0.01]
Deng HL 2012	33	14.61	74	31.73	14.98	177	14.4%	0.09 [-0.19, 0.36]
Du H 2017	40.57	19.58	42	50.69	35.29	38	10.0%	-0.36 [-0.80, 0.09]
Jiang YH 2016	39.48	12.07	30	38.95	12.87	58	10.0%	0.04 [-0.40, 0.48]
Niu HY 2011	45.2	8.7	25	46.1	7.9	25	7.8%	-0.11 [-0.66, 0.45]
Song N 2014	48.7	38.3	75	63.5	28.8	33	10.7%	-0.41 [-0.82, 0.00]
Sun YL 2012	57.18	31.58	132	56.53	29.98	74	14.1%	0.02 [-0.26, 0.31]
Wang F 2017	40.11	3.86	102	42.23	2.76	80	13.6%	-0.62 [-0.92, -0.32]
Zhang QF 2015	56.3	34.52	25	53.74	38.16	119	10.2%	0.07 [-0.36, 0.50]
**Total (95% CI)**			**528**			**671**	**100.0%**	**-0.19 [-0.39, 0.01]**

Heterogeneity: Tau² = 0.05; Chi² = 19.43, df = 8 (P = 0.01); I² = 59%

Test for overall effect: Z = 1.87 (P = 0.06)

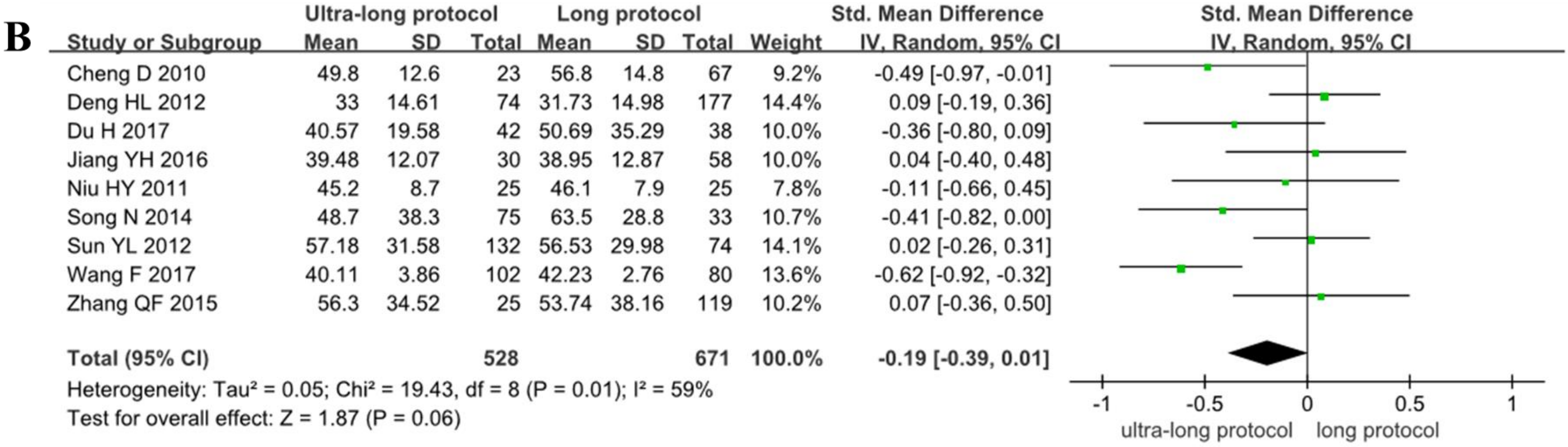

C

Study or Subgroup	Ultra-long protocol Mean	SD	Total	Short protocol Mean	SD	Total	Weight	Std. Mean Difference IV, Random, 95% CI
Cheng D 2010	49.8	12.6	23	54.3	13.3	49	18.5%	-0.34 [-0.84, 0.16]
Du H 2017	40.57	19.58	42	53.34	29.85	40	20.6%	-0.50 [-0.94, -0.06]
Jiang YH 2016	39.48	12.07	30	38.9	11.47	38	19.2%	0.05 [-0.43, 0.53]
Sun YL 2012	57.18	31.58	132	48.52	30.53	56	25.7%	0.28 [-0.04, 0.59]
Zhang QF 2015	56.3	34.52	25	54.41	35.68	21	15.9%	0.05 [-0.53, 0.63]
**Total (95% CI)**			**252**			**204**	**100.0%**	**-0.08 [-0.39, 0.24]**

Heterogeneity: Tau² = 0.07; Chi² = 9.76, df = 4 (P = 0.04); I² = 59%

Test for overall effect: Z = 0.49 (P = 0.63)

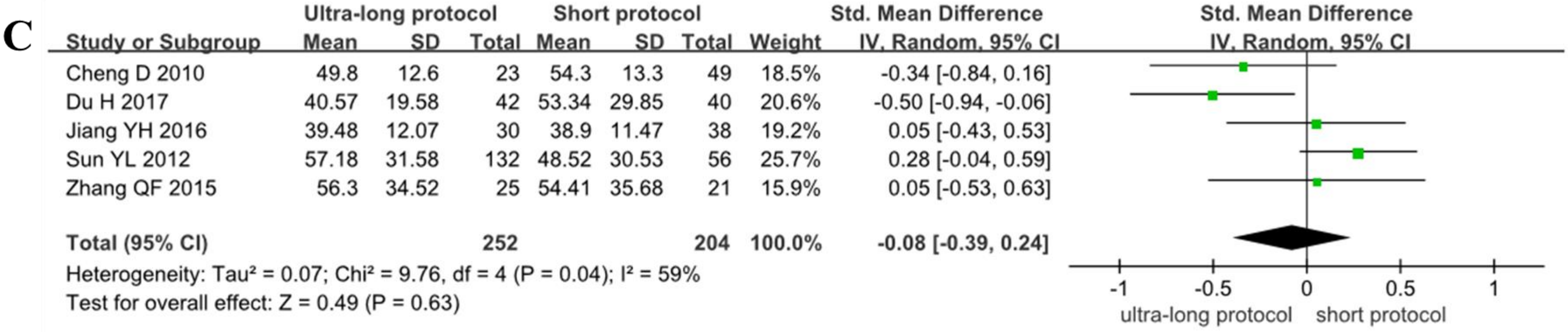

Fig. S4 Meta-analysis on basal E2 levels: the ultra-long protocol versus long protocol in RCTs (A) and in non-RCTs (B); the ultra-long protocol versus short protocol in non-RCTs (C).
