# Supplementary material for: The effectiveness of different down-regulating protocols on in vitro fertilization-embryo transfer in endometriosis: a meta-analysis
Source: Reprod Biol Endocrinol. 2020 Feb 29;18:16. doi: 10.1186/s12958-020-00571-6 (PMC7049222; doi:10.1186/s12958-020-00571-6)
Supplement: Supplementary file 9 — Additional file 9: Figure S5. Meta-analysis on basal LH levels in non-RCTs: the ultra-long protocol versus long protocol (A), and the ultra-long protocol versus short protocol (B). [file 12958_2020_571_MOESM9_ESM.pdf]

A

Study or Subgroup	Ultra-long protocol Mean	SD	Total	Long protocol Mean	SD	Total	Weight	Std. Mean Difference IV, Random, 95% CI
Deng HL 2012	3.86	2.81	74	3.32	1.19	177	25.5%	0.30 [0.02, 0.57]
Du H 2017	5.74	8.41	42	3.23	0.97	38	16.0%	0.41 [-0.04, 0.85]
Song N 2014	5.28	5.9	75	4.38	1.9	33	17.5%	0.18 [-0.23, 0.59]
Sun YL 2012	4.77	2.52	132	4.54	2	74	24.6%	0.10 [-0.19, 0.38]
Zhang QF 2015	4.71	2.53	25	5.51	2	119	16.4%	-0.38 [-0.81, 0.05]
**Total (95% CI)**			**348**			**441**	**100.0%**	**0.13 [-0.10, 0.36]**

Heterogeneity: Tau² = 0.03; Chi² = 8.24, df = 4 (P = 0.08); I² = 51%
Test for overall effect: Z = 1.14 (P = 0.26)

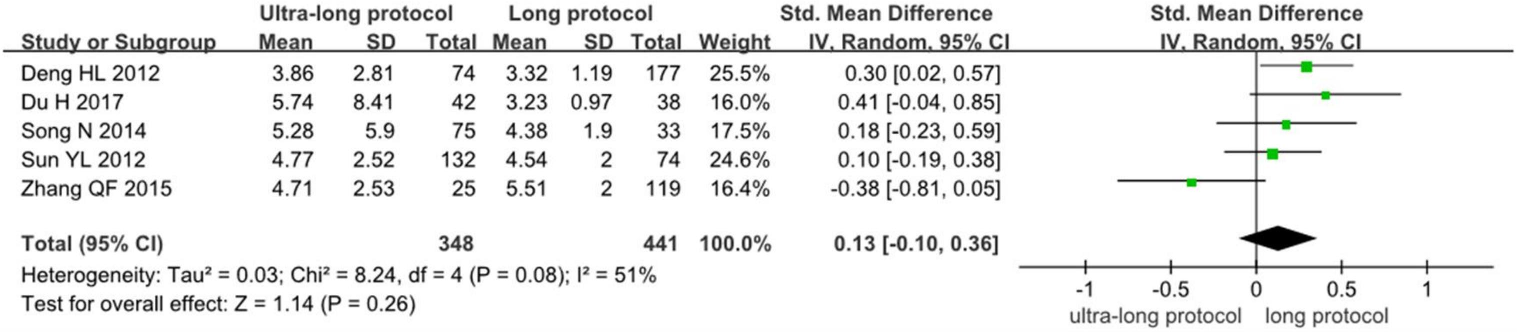

B

Study or Subgroup	Ultra-long protocol Mean	SD	Total	Short protocol Mean	SD	Total	Weight	Std. Mean Difference IV, Fixed, 95% CI
Du H 2017	5.74	8.41	42	4.42	1.71	40	28.7%	0.21 [-0.22, 0.65]
Sun YL 2012	4.77	2.52	132	4.93	3	56	55.3%	-0.06 [-0.37, 0.25]
Zhang QF 2015	4.71	2.53	25	4.8	2.23	21	16.1%	-0.04 [-0.62, 0.54]
**Total (95% CI)**			**199**			**117**	**100.0%**	**0.02 [-0.21, 0.25]**

Heterogeneity: Chi² = 1.04, df = 2 (P = 0.59); I² = 0%
Test for overall effect: Z = 0.19 (P = 0.85)

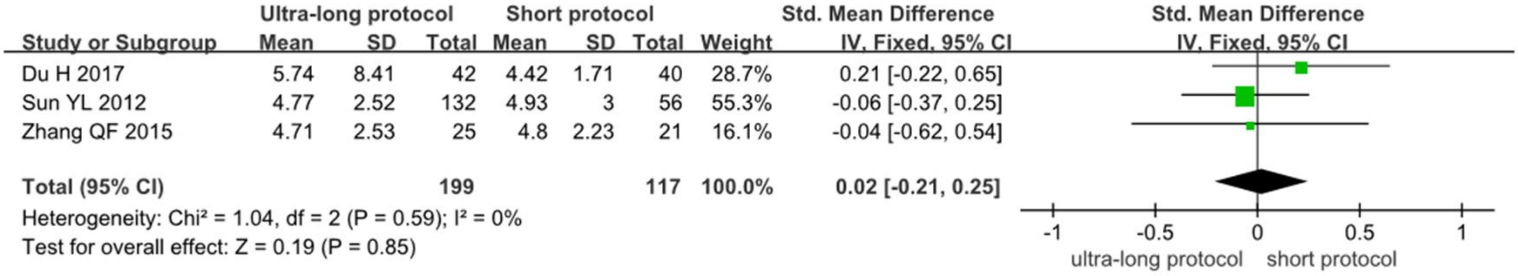

Fig. S5 Meta-analysis on basal LH levels in non-RCTs: the ultra-long protocol versus long protocol (A), and the ultra-long protocol versus short protocol (B).
