# Supplementary material for: The effectiveness of different down-regulating protocols on in vitro fertilization-embryo transfer in endometriosis: a meta-analysis
Source: Reprod Biol Endocrinol. 2020 Feb 29;18:16. doi: 10.1186/s12958-020-00571-6 (PMC7049222; doi:10.1186/s12958-020-00571-6)
Supplement: Supplementary file 10 — Additional file 10: Figure S6. Meta-analysis on CA125 in non-RCTs: the ultra-long protocol versus long protocol before down-regulation (A), and after down-regulation (B). [file 12958_2020_571_MOESM10_ESM.pdf]

A

Study or Subgroup	Ultra-long protocol Mean	SD	Total	Long protocol Mean	SD	Total	Weight	Std. Mean Difference IV, Random, 95% CI
Deng HL 2012	53.25	41.57	74	21.43	12.74	177	50.1%	1.27 [0.98, 1.57]
Wang F 2017	39.59	12.16	102	31.03	20	80	49.9%	0.53 [0.23, 0.83]
**Total (95% CI)**			**176**			**257**	**100.0%**	**0.90 [0.17, 1.63]**

Heterogeneity: Tau² = 0.25; Chi² = 12.11, df = 1 (P = 0.0005); I² = 92%
Test for overall effect: Z = 2.43 (P = 0.02)

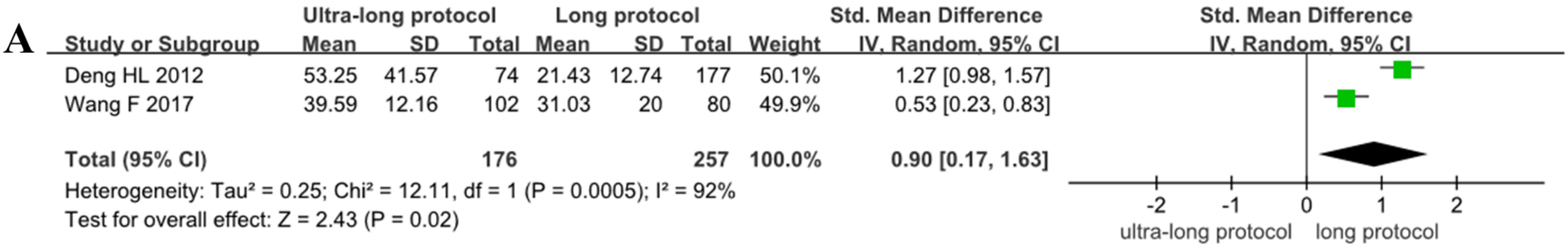

B

Study or Subgroup	Ultra-long protocol Mean	SD	Total	Long protocol Mean	SD	Total	Weight	Std. Mean Difference IV, Random, 95% CI
Deng HL 2012	15.89	7.18	74	18.75	8.21	177	50.4%	-0.36 [-0.63, -0.09]
Wang F 2017	35.57	10.17	102	31.93	17	80	49.6%	0.27 [-0.03, 0.56]
**Total (95% CI)**			**176**			**257**	**100.0%**	**-0.05 [-0.66, 0.56]**

Heterogeneity: Tau² = 0.18; Chi² = 9.36, df = 1 (P = 0.002); I² = 89%
Test for overall effect: Z = 0.16 (P = 0.88)

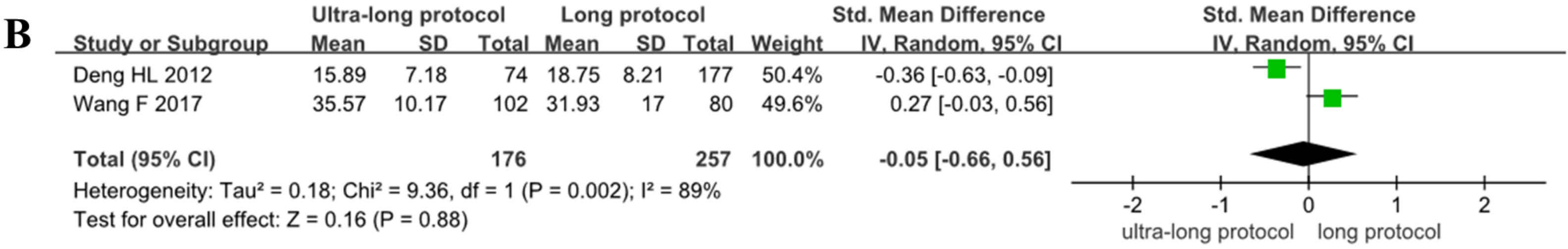

Fig. S6 Meta-analysis on CA125 in non-RCTs: the ultra-long protocol versus long protocol before down-regulation (A), and after down-regulation (B).
